# The Effects of Maternal Separation on Adult Methamphetamine Self-Administration, Extinction, Reinstatement, and MeCP2 Immunoreactivity in the Nucleus Accumbens

**DOI:** 10.3389/fpsyt.2013.00055

**Published:** 2013-06-17

**Authors:** Candace R. Lewis, Kelsey Staudinger, Lena Scheck, M. Foster Olive

**Affiliations:** ^1^Department of Psychology, Arizona State UniversityTempe, AZ, USA

**Keywords:** maternal separation, Mecp2, methamphetamine, early life stress, self-administration, nucleus accumbens, epigenetics

## Abstract

The maternal separation (MS) paradigm is an animal model of early life stress. Animals subjected to MS during the first 2 weeks of life display altered behavioral and neuroendocrinological stress responses as adults. MS also produces altered responsiveness to and self-administration (SA) of various drugs of abuse including cocaine, ethanol, and amphetamine. However, no studies have yet examined the effects of MS on methamphetamine (METH) SA. This study was performed to examine the effects of MS on the acquisition of METH SA, extinction, and reinstatement of METH-seeking behavior in adulthood. Given the known influence of early life stress and drug exposure on epigenetic processes, we also investigated group differences in levels of the epigenetic marker methyl CpG binding protein 2 (MeCP2) in the nucleus accumbens (NAc) core. Long–Evans pups and dams were separated on postnatal days (PND) 2–14 for either 180 (MS180) or 15 min (MS15). Male offspring were allowed to acquire METH SA (0.05 mg/kg/infusion) in 15 2-h daily sessions starting at PND67, followed by extinction training and cue-induced reinstatement of METH-seeking behavior. Rats were then assessed for MeCP2 levels in the NAc core by immunohistochemistry. The MS180 group self-administered significantly more METH and acquired SA earlier than the MS15 group. No group differences in extinction or cue-induced reinstatement were observed. MS15 rats had significantly elevated MeCP2-immunoreactive cells in the NAc core as compared to MS180 rats. Together, these data suggest that MS has lasting influences on METH SA as well as epigenetic processes in the brain reward circuitry.

## Introduction

Methamphetamine (METH) is an extremely potent and highly addictive psychostimulant and neurotoxic drug (Xie and Miller, 2009). METH abuse has many detrimental consequences for the individual and for society as a whole. For the individual, chronic abuse has negative neuropsychological and psychiatric effects, as well as modifying the healthy brain’s functional and structural reward and learning neurocircuitry (Darke et al., 2008; Krasnova and Cadet, 2009; Taylor et al., 2013). METH abuse has been identified as both a strong risk factor for violence and high-risk sexual behaviors. In one study of a population between the ages of 18 and 25, 34.9% self-reported violent behavior while under the influence of METH, such as domestic violence, gang-related violence, and random acts of violence (Sommers et al., 2006). Individuals on METH often engage in unprotected vaginal and anal sex and also have sex with multiple partners (Springer et al., 2007). It is apparent that chronic METH use has a multitude of deleterious effects on both the users and society as a whole.

Since METH use has been associated with a variety of negative health and social consequences, it is important to identify risk-factors associated with its abuse. Clinical research has shown early life stress, particularly childhood abuse and neglect, is a reliable risk factor that influences adult drug abuse (Anda et al., 2006; Messina et al., 2008). Childhood abuse or neglect is highly prevalent with ∼1.5 million cases reported in 2010 (Child Maltreatment 2010, U.S. Department of Health and Human Services) and exposure to childhood abuse and household dysfunction has been related to an earlier onset of METH use in both men and women (Messina et al., 2008). There is substantial evidence that early life stress produces long-lasting changes in the brain, including regions that mediate reward-seeking and executive control, which may ultimately predispose the individual to increased propensity toward illicit drug use and addiction (Matthews et al., 2001; Meaney et al., 2002). Stressors during adulthood have also been implicated in affecting drug and alcohol self-administration (SA) (Piazza et al., 1990; Breese et al., 2011).

The rodent maternal separation (MS) model of early life stress is a commonly used paradigm to investigate the influences of early life events on addictive behaviors. In this paradigm, rodents undergo daily separation from maternal care during critical postnatal development and later assessed for propensity toward addiction-like behaviors in adulthood. For example, pups undergoing MS for several hours exhibit depression-like symptoms, high anxiety-like behavior, exaggerated neuroendocrinological responses to stress, and have a high preference for ethanol (Huot et al., 2001). MS has been reported to also alter the reinforcing effects of cocaine, amphetamine, and morphine (Vazquez et al., 2005; Moffett et al., 2006; Der-Avakian and Markou, 2010). However, only a few reports have been published on the effects of MS on adult METH-seeking behavior. In one study, MS failed to produce a significant increase in adolescent METH conditioned place preference (CPP) (Faure et al., 2009), while another study demonstrated that MS attenuated METH CPP in adolescents (Dimatelis et al., 2012a). MS has also been shown to produce a sex- and dose-dependent increase in locomotor and stereotypy responses to METH in adolescent rats (Pritchard et al., 2012). To our knowledge, however, there are no reports to date on the effect of MS on adult intravenous (i.v.) METH SA, extinction, and reinstatement. Given the negative impact of METH abuse and the relationship observed between MS and other drugs of abuse, more research in this area is warranted.

Emerging evidence suggests a strong role of epigenetics in regulating gene transcription based on early experiences that in turn modulate brain systems and behavior into adulthood. Many neural systems implicated in drug addiction are influenced by MS, such as the hypothalamic pituitary adrenal (HPA) axis (Plotsky and Meaney, 1993), endocannabinoid system (Romano-López et al., 2012), monoaminergic systems (Matthews et al., 2001; Ploj et al., 2003; Dimatelis et al., 2012b), and growth factors such as BDNF (Bolaños and Nestler, 2004; Lippmann et al., 2007). Recent studies implicate epigenetic modifications as a mechanism behind these changes in rodents, non-human primates, and humans (McGowan et al., 2009; Murgatroyd et al., 2009; Kinnally et al., 2011). For example, Murgatroyd et al. (2009) showed that MS induced hypomethylation of the arginine-vasopressin (Avp) enhancer, subsequently causing upregulation of Avp expression and a hyper-responsive HPA axis. Additionally, maternal care has been implicated in DNA methylation and corresponding changes in glucocorticoid receptor (GR) expression levels in the hippocampus (Weaver et al., 2004). Furthermore, adult rats exposed to early life stress have demonstrated reduced BDNF in the prefrontal cortex correlated with hypermethylation of the BDNF IV promoter region (Roth et al., 2009). Indeed, these studies suggest that early life experiences are influencing epigenetic markers that modulate multiple brain systems implicated in drug vulnerability.

Interestingly, epigenetic factors are also altered by drug exposure and can influence drug intake, behavioral, and neural responses (Renthal and Nestler, 2008; Robison and Nestler, 2011; Lewis and Olive, in press). For example, trimethylation of histone H3 lysine 4 (H3K4) at the promoter region of a chemokine receptor type 2 (CCR2), a gene implicated in locomotor sensitization, has been associated with METH-induced hyperlocomotion in mice (Ikegami et al., 2010). Additionally, cocaine increases methyl CpG binding protein 2 (MeCP2) expression in multiple brain regions of the rat (Cassel et al., 2006) and MeCP2 has been implicated in cocaine and amphetamine reward and reinforcement (Deng et al., 2010; Im et al., 2010). Specifically, Deng et al. (2010) found that virally mediated ablation of MeCP2 expression in the nucleus accumbens (NAc) increased the conditioned rewarding effects of amphetamines, whereas overexpression of MeCP2 in the NAc decreased amphetamine reward. Furthermore, Im et al. (2010) showed that cocaine intake was reduced after knockdown of MeCP2 expression in the dorsal striatum. Hence, recent studies suggest that the predisposition of one’s epigenetic phenotype may influence their behavioral response to drugs of abuse while exposure to drugs of abuse also modulates their epigenetic phenotype.

Although MS and psychostimulants have been shown to individually affect epigenetic factors such as MeCP2, and MeCP2 has been implicated in drug seeking behavior, it is yet to be determined if MS and, specifically, METH also interact to affect epigenetic factors. Therefore, the goal of the present study was to investigate the relationship between early life stress, METH SA, extinction and reinstatement, and MeCP2 expression in the NAc. We hypothesized that MS would increase susceptibility to the acquisition of METH SA, impair extinction learning, and increase cue-induced reinstatement. We also predicted that MeCP2 expression in the NAc would be negatively correlated with levels of METH SA.

## Materials and Methods

All experimental and surgical procedures were carried out in adherence to the National Institutes of Health *Guide for the Care and Use of Laboratory Animals* (National Research Council, 1996) and approved by the Institutional Animal Care and Use Committee of Arizona State University.

### Animals and maternal separation procedures

Pregnant dams were purchased from Charles River Laboratories and arrived on gestational day 12 (GD12). Dams were housed individually in standard polycarbonate cages in a temperature and humidity controlled room with food and water available *ad libitum*. Beginning on GD20 (range of gestation 21–23 days) cages were checked for delivery of pups three times a day. Litters were culled to a maximum size of 12 immediately after discovery. Litter sizes ranged from 10 to 12 with one litter at eight due to pup attrition. The litter sex ratios were left natural with an average of 7/6 male/female ratio across all litters. Day of birth was considered postnatal day 0 (PND0).

Litters were randomly assigned to one of two conditions: MS for 180 min per day (MS180) or the handled group, 15 min per day (MS15). After pup attrition due to filicide, and exclusion of animals that lost catheter patency during the experiment, the MS15 (*n* = 9) condition had three litters with one to four male pups per litter and the MS180 (*n* = 17) group had five litters with two to five male pups per litter that were used in the behavioral testing. The separation procedure began on PND2. At 8:00 a.m. (reverse light cycle, lights off at 7:00 a.m.) the dam was removed from the home cage and placed into a new cage with fresh bedding. The pups were then removed and placed into a separation cage kept in an isolated room. Heat lamps were set over the separation cages and maintained at 30 ± 0.5 to 32 ± 0.5 °C to control for hypothermic conditions. The pups were left unattended during the corresponding separation period then returned to the home-cage immediately prior to the dams return.

During PND15–19 litters were left undisturbed, weaned on PND21 into same sex group housing, and pair housed with a sibling on PND45. After separation procedures rats were left undisturbed with the exception of once a week cage cleaning performed by Department of Animal Care and Treatment employes. Females were not used for the remainder of the study.

### Surgical procedures

Male rats were implanted with i.v. catheters into the jugular vein on PND60 ± 1 day. Rats were anesthetized with isoflurane (2% v/v, Butler Schein Animal Health, Dublin, OH, USA) vaporized in oxygen at a flow rate of 2 l/min. Rats received pre-incision injections of buprenorphine (0.05 mg/kg, s.c., Reckitt Benckiser, Richmond, VA, USA) and meloxicam (1 mg/kg, s.c., Boehringer Ingelheim, St. Joseph, MO, USA). Surgical sites were shaved and cleaned with 1% iodine. A ∼2 cm incision was made in order to isolate the right or left jugular vein. A sterile silastic catheter filled with 100 U/ml heparin was inserted 2.5 cm into the vein. The catheter was secured to the surrounding tissue with sutures, and the opposite end of the catheter was tunneled subcutaneously to the dorsum where it exited the skin between the scapulae. The catheter was secured to the surrounding tissue by sutures and a mesh collar attached to a threaded vascular access port (Plastics One, Roanoke, VA, USA). The wound was then treated with 0.2 ml bupivacaine hydrochloride (0.25% v/v), closed with nylon sutures (Ethicon, San Lorenzo, Puerto Rico) and topically treated with topical lidocaine and a triple antibiotic gel. The access port was sealed with a piece of Tygon tubing closed at one end and a threaded protective cap (Plastics One). Rats were given small portions of sweetened cereal to facilitate postsurgical rehabilitation. Following surgical procedures, rats were allowed at least 7 days of recovery and received daily i.v. infusions of 0.2 ml Timentin and 0.2 ml heparin to minimize infections and maintain catheter patency.

### Self-administration apparatus

Behavioral testing was conducted in SA chambers (ENV-008; Med Associates Inc., St. Albans, VT, USA) that were interfaced to a PC computer and located in sound attenuating melamine enclosures equipped with ventilation fans. The chambers (28 cm × 27 cm × 22 cm) consisted of two aluminum walls and two clear Plexiglas walls. The ceiling was also constructed of Plexiglas with a 3-cm diameter hole cut in the center to allow a drug delivery tether to pass through. The floor consisted parallel stainless steel rods (0.48 cm diameter) placed 1.6 cm apart. Each chamber contained a house light located 1.25 cm from the ceiling, a Sonalert speaker that provided an auditory stimulus (∼65 dB, 2900 Hz) during drug infusion, one retractable response lever, one stationary response lever, and two 2.5 cm stimulus cue lights located above each response lever. The retractable lever was designated the active lever as an additional cue for drug availability. Response levers were located 7 cm above the floor of the chamber. Centered between the levers was a 5 cm × 5 cm food pellet receptacle. Each chamber was outfitted with a single-speed automated drug infusion pump (PHM-100; Med Associates). Tygon microbore tubing (0.5 mm ID) was used to connect the syringe containing the drug solution to a single-channel liquid swivel that was mounted to the top of the chamber enclosure. The swivel was then connected to the vascular access port using Tygon microbore tubing that was protected by a stainless steel tether (Plastics One, Roanoke, VA, USA). All experimental parameters were controlled using Med PC IV software (Med Associates).

### Methamphetamine self-administration, extinction, and cue-induced reinstatement

Beginning on PND67 male rats underwent 2 h daily SA sessions whereby presses on one of the levers (designated the active lever) resulted in delivery of METH (0.1 mg/kg per infusion, delivered in a volume of 0.06 ml over a 2-s period) on an fixed ratio 1 (FR1) schedule of reinforcement. Each METH infusion delivery was followed by a 20-s timeout period, during which additional active lever presses were recorded but produced no drug infusions. Each infusion was accompanied by concurrent illumination of a stimulus light located directly above the active lever, and presentation of an auditory stimulus for 2 s. SA sessions were conducted 7 days per week for 15 consecutive days. METH hydrochloride (Sigma Aldrich, St. Louis, MO, USA) was dissolved in 0.9% sterile saline for i.v. SA.

Next, all animals were subjected to extinction training, whereby presses on the active lever no longer produced any programed consequences (i.e., no tone/light presentation and no activation of the syringe pump). Extinction training sessions were 2 h in length and were conducted for 15 consecutive days. On the day immediately following the last extinction session, all rats underwent cue-induced reinstatement, whereby presses on the active lever produced the tone and light cue previously presented during METH infusion, but did not deliver any drug solution. Presses on the inactive lever did not produce any programed consequences throughout the experiment.

### Tissue preparation and immunohistochemistry

Immunochemistry procedures were carried out according to standard procedures. Brain tissues were collected on the day following the reinstatement test session. Rats were deeply anesthetized with 150 mg/kg i.p. sodium pentobarbital and perfused transcardially with ice-cold 0.1 M phosphate buffered saline (PBS) followed by ice-cold 4% w/v paraformaldehyde (PFA) in PBS, pH 7.4. Brains were removed, post-fixed in 4% PFA overnight and stored in 30% w/v sucrose in PBS. Brains were sectioned (35 μm thickness) in the coronal plane on a cryostat (Leica CM1900, Bannockburn, IL, USA). Sections were then rinsed 3 × 10 min in PBS containing 0.1% v/v Tween 20 (PBST) followed by incubation in PBST containing 5% v/v normal donkey serum for 1 h. Sections were then incubated overnight under gentile agitation at 4° C in PBST containing a rabbit anti-MeCP2 polyclonal antibody (PA1-887; 1:200 dilution; Thermo Scientific) and then rinsed 3 × 10 min in PBS. Sections were then incubated in PBS containing Alexa Fluor 488 conjugated donkey anti-rabbit IgG antisera (1:200; Jackson ImmunoResearch, West Grove, PA, USA) and then rinsed 3 × 10 min in PBS. Sections were mounted on microscope slides using VectaShield mounting media (Vector Labs, Burlingame, CA, USA), coverslipped, and stored in darkness until imaging.

### Immunoreactivity analysis

Investigator was blind to treatment condition during microscopical analysis. Sections were visualized at 200× magnification using a Leica DMLB epifluorescence microscope equipped with a digital camera that was interfaced to a PC. Digital images of the selected area were obtained using Leica IM50 software and counted by two observers blind to treatment conditions using the ImageJ Tool software package (Rasband, W.S., ImageJ, U.S. National Institutes of Health, Bethesda, MD, USA). The average background removed by the software was 50. A total of six sample areas of the NAc were counted for each subject (i.e., one sample area/two hemispheres/three sections). NAc core area was chosen based on the corpus callosum as a landmark. Care was taken to ensure that the sections for each subject that were labeled came from the same anatomical level within each plane. The counts from all six sample areas from a particular region were averaged to provide a mean number of immunoreactive cells per animal to be used as an *n* = 1 for statistical analysis (Thiel et al., 2009). Inter rater reliability was 89%.

### Data analysis

The alpha level was set at 0.05 for all statistical analyses and analyzed using IBM SPSS Statistics 20 software. A repeated-measures ANCOVA with litter as the factor and rearing condition as the covariate was used to test for litter effects. Separate repeated-measures ANOVAs with rearing condition as a between-subjects factor and session as a within-subjects factor were used to analyze active and inactive lever presses during SA and extinction. The correlation between METH-seeking behavior and MeCP2 expression within the NAc was calculated using Pearson’s product correlation.

## Results

### Litter effects

A repeated-measures ANCOVA was conducted by litters and controlling for rearing condition on the number of METH infusions per session over 15 days revealed no significant pre-existing differences between litters (*p* = 0.23).

### METH self-administration

**Figure 1:**
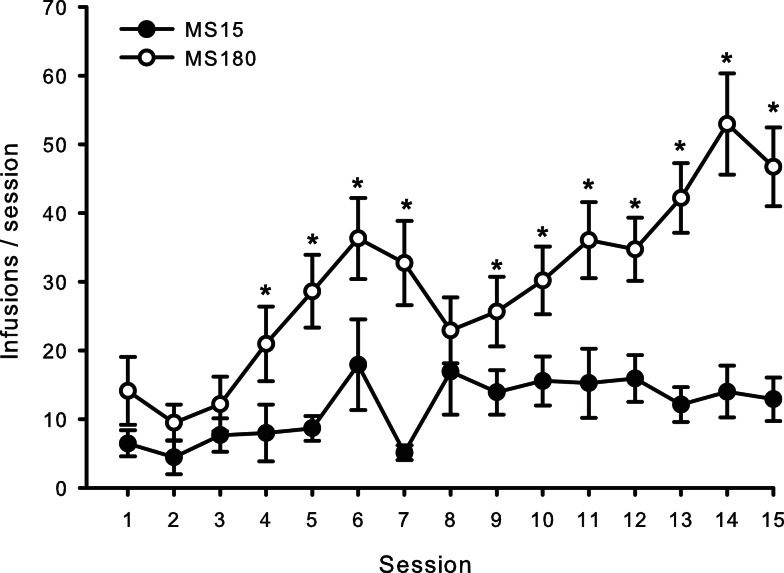
**Average number of METH SA infusions per 2-h session for 15 consecutive days in MS15 (*n* = 9) and MS180 (*n* = 17) rats**. Data points represent group mean ± SEM. *p < 0.05 vs. MS15.

**Figure 2:**
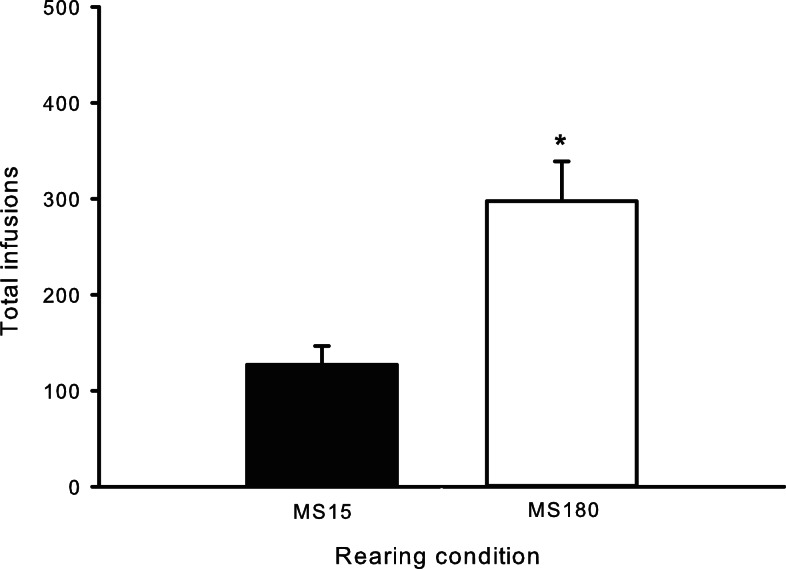
**Total number of METH infusions earned across 15 daily 2-h sessions in MS15 (*n* = 9) and MS180 (*n* = 17) rats**. Data points represent group mean ± SEM. *p < 0.05 vs. MS15.

A total of *n* = 4 animals were removed from the MS15 and MS180 groups respectively due to loss of catheter patency. Repeated-measures ANOVA revealed a significant main effect of rearing condition on the number of METH infusions per session [*F*(1,24) = 9.83, *p* = 0.004] (see Figures 1 and 2), as well as the number of total active lever presses per session [*F*(1,24) = 13.79, *p* = 0.001], MS180 had more active lever presses and received more infusions than MS15. No group differences in the total number of inactive lever presses were observed [*F*(1,17) = 38.76, *p* = 0.425]. However, in both rearing conditions we noted a time-dependent increase in inactive lever pressing across SA sessions (see Table 1), and we attribute this to be a result of non-specific motor activity that resulted from increasing level of METH SA.

**Table 1:** **Mean ± SEM active or inactive lever presses across 15 METH SA sessions (in 5 session bins), 15 extinction sessions, and the cue-induced reinstatement session**.

	MS15	MS180
**SELF-ADMINISTRATION**		
Active lever presses (sessions 1–5)	35 ± 11	85 ± 15
Active lever presses (sessions 6–10)	51 ± 11	111 ± 19
Active lever presses (sessions 11–15)	70 ± 14	213 ± 23
Inactive lever presses (sessions 1–5)	122 ± 46	73 ± 18
Inactive lever presses (sessions 6–10)	97 ± 25	101 ± 25
Inactive lever presses (sessions 11–15)	144 ± 36	167 ± 36
**EXTINCTION**		
Active lever presses	147 ± 22	151 ± 10
Inactive lever presses	302 ± 100	117 ± 16*
**REINSTATEMENT**		
Active lever presses	18 ± 4	24 ± 3
Inactive lever presses	7 ± 3	8 ± 2

*Indicates p < 0.05 vs. inactive lever presses during extinction in the MS15 group.

### Extinction

**Figure 3:**
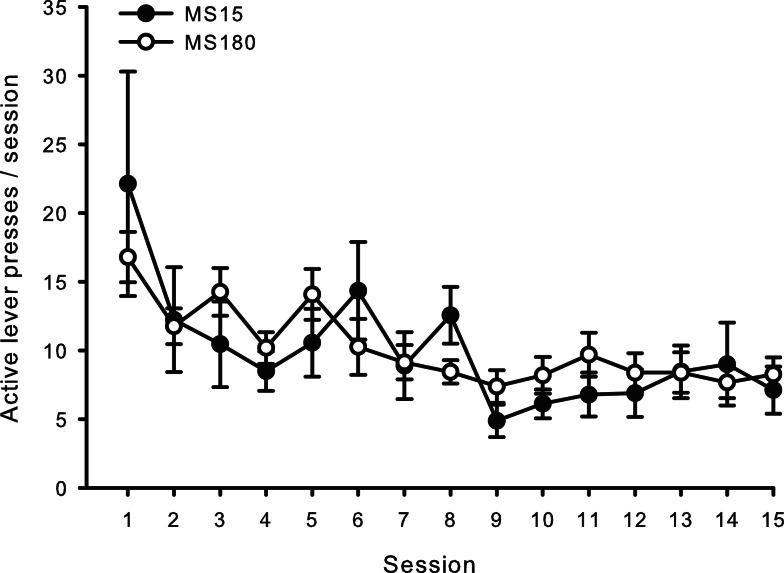
**Average number of active lever presses per 2-h session for 15 consecutive days during extinction in MS15 (*n* = 9) and MS180 (*n* = 17) rats**. Data points represent group mean ± SEM. No significant differences between rearing conditions were observed.

For both groups, extinction training produced a significant reduction in the number of active lever presses when comparing the average of the final 2 days of Ext to the average of the final 2 days of SA (*n* = 26) [*t*(50) = 5.10, *p* < 0.0001]. Repeated-measures ANOVA revealed no significant group differences (MS15 *n* = 9, MS180 *n* = 17) in rate of extinction of active lever pressing [*F*(1,20) = 0.94, *p* = 0.34] (see Figure 3). However, a significant group difference in the number of inactive lever presses during extinction training [*F*(1,24) = 5.47, *p* = 0.028] was observed, with rats in the MS15 group emitting more inactive lever presses over the 15-day extinction period compared to the MS180 group (see Table 1).

### Cue-induced reinstatement

**Figure 4:**
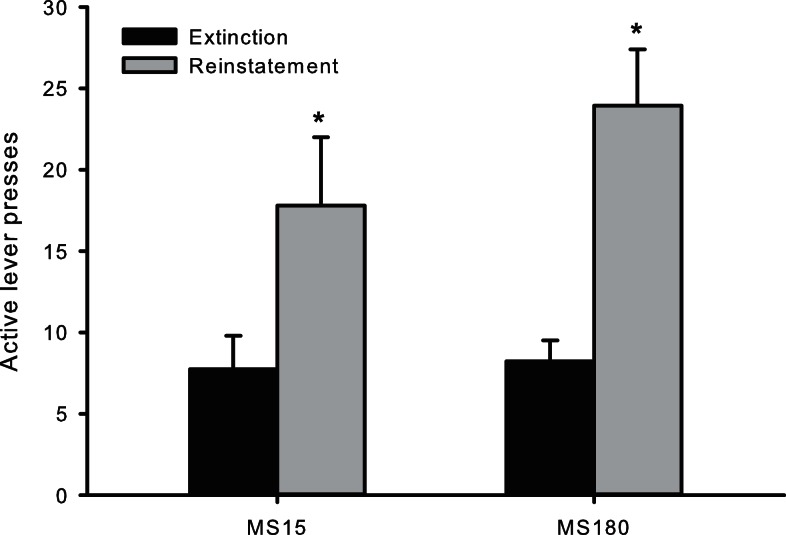
**Number of active lever presses across the final 2 days of extinction training and during cue-induced reinstatement in MS15 (*n* = 9) and MS180 (*n* = 17) rats**. Data points represent group mean ± SEM. *p < 0.05 vs. extinction.

Cue-induced reinstatement was observed in both groups as assessed by the number of active lever presses (averaged across the final 2 days of Ext) compared to active lever presses during the reinstatement session [*t*(50) = −4.46, *p* < 0.0001]. However, there was no significant difference between the groups for the number of active lever presses during reinstatement testing [*F*(1,24) 1.134, *p* = 0.298] (see Figure 4).

### MeCP2 immunoreactivity

**Figure 5:**
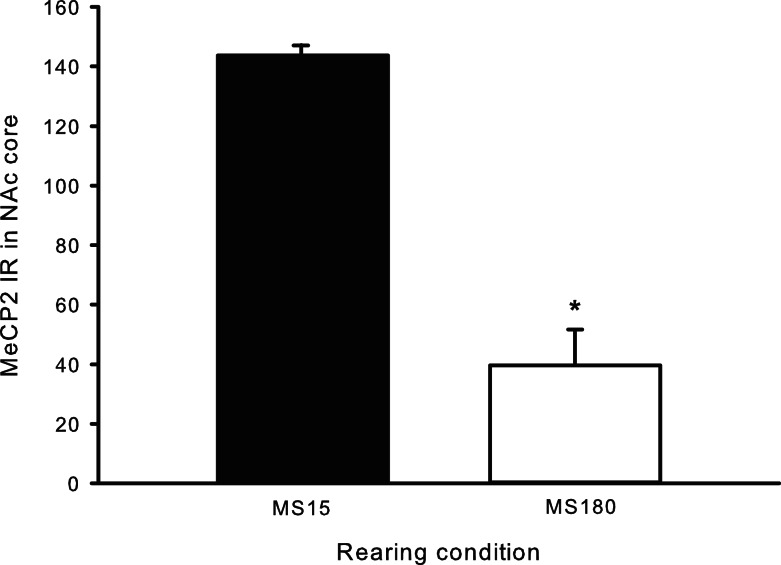
**Cell counts for MeCP2 immunoreactivity in the NAc core for MS15 (*n* = 5) and MS180 (*n* = 5) rats**. Data points represent group mean ± SEM. *p < 0.05 vs. MS15.

**Figure 6:**
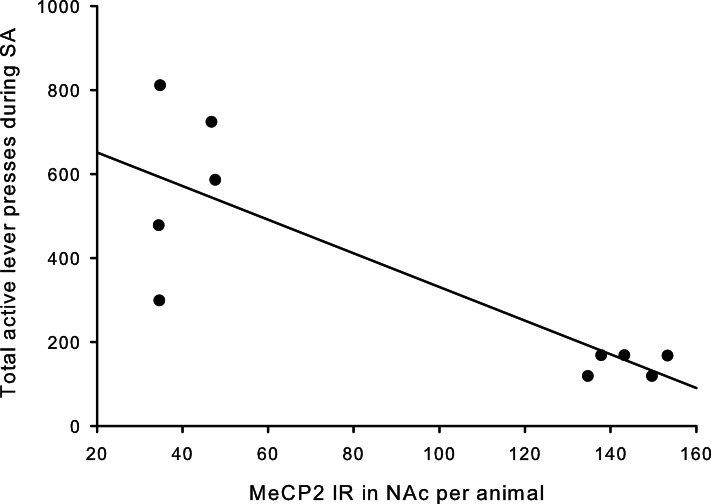
**Number of active lever presses negatively correlated with MeCP2 immunoreactivity in the NAc core (*r* = −0.836, *p* = 0.003)**.

**Figure 7:**
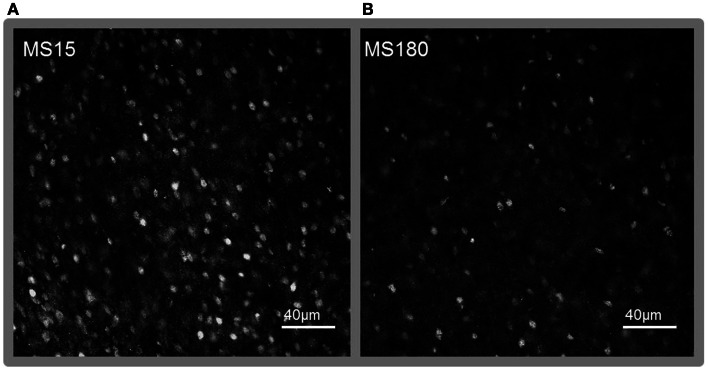
**Representative photomicrographs of immunolabeling for MeCP2 in the NAc core**. **(A)** MS15, **(B)** MS180. Scale bar represents 40 μm.

A total of ten pups from five different litters (three per rearing condition) were used in the analysis of the MeCP2 data. There was a highly significant difference in MeCP2 immunoreactivity between MS15 rats and MS180 rats in the NAc core, *p* < 0.001, with MS15 expressing more labeled profiles than did MS180 (see Figure 5). There was also a negative correlation between MeCP2 immunoreactivity and number of total active lever presses during 15 days of SA, *r* = −0.839, *p* = 0.003 (*n* = 5 per rearing condition) (see Figure 6). Rats emitting fewer lever presses expressed higher numbers of labeled profiles in the NAc core (Figure 7).

## Discussion

Early life maternal care is known to influence a multitude of neurological, endocrine, epigenetic, and behavioral outcomes in adulthood (Francis et al., 1999; Roth, 2012). Our findings contribute to the literature by suggesting that MS causes alterations that influence vulnerability to drug abuse (Moffett et al., 2007), in this case METH SA. For the first time, our study suggests that either repeated and prolonged MS leads to increased vulnerability to METH intake or that minimal MS protects against adult METH SA vulnerability. This is evidenced by our findings that MS180 rats showed higher levels of METH SA over 15 daily sessions compared to MS15. These findings are in agreement with previous studies examining effects of MS on intake of cocaine, morphine, amphetamine, and ethanol (Huot et al., 2001; Vazquez et al., 2005; Moffett et al., 2006; Der-Avakian and Markou, 2010). Additionally, we noted that MS15 rats demonstrated a preference for the inactive lever over the active lever during SA. While the reason for this is currently unknown, a possible explanation for this counterintuitive observation is different non-specific behavioral response to METH SA or enhanced operant sensation seeking in the MS15 group.

The possible protective or resilient effect in the MS15 group provides an interesting comparison. MS15 adults have shown reduced responding for cocaine when compared to non-separated controls (Flagel et al., 2003; Moffett et al., 2006). Since our data set does not include a non-handled control it is difficult to distinguish whether the SA behavior is reflective of increased vulnerability in the MS180, protective effects in MS15, or both, but the robust differences are clear. The effects of brief and prolonged MS we found on METH SA fits the inverted U-shape resilience function usually found in drug abuse-related behaviors after MS (Neisewander et al., 2012). It has been postulated that the protective effects seen in the MS15 group may be due to the increased maternal care post separation (Marmendal et al., 2004; Francis and Kuhar, 2008). Many have argued that the MS15 rearing condition is more ethologically relevant than the standard non-separated controls since food foraging and other activities would necessitate the dam to leave the litter for brief amounts of time.

The current literature on MS and drug reward, reinforcement, and SA demonstrates that MS180 and MS15 tend to be the most divergent groups when compared to the various controls. For this reason, in the present study, we did not include a non-separated control group in order to increase validity and reliability in our data and improve interpretation in comparison with other studies. Additionally, there are large inconsistencies across laboratories with regards to procedures for breeding, culling, fostering, litter sex ratios, separation duration and days, the order in which dams and pups are returned to the home cage, controlled temperature settings outside of the home cage, PND of weaning, and post-weaning housing conditions prior to and during manipulations. Furthermore, the use of control groups (including MS0, non-handled, and Animal Facility Reared) is highly variable. The issues concerning different control groups and variations in procedures have previously been discussed by others (Matthews et al., 1999, 2001). Jaworski et al. (2005) provides a well laid out table comparing different experimental and control groups commonly used. Recently, a trend toward comparing only two groups has emerged. For example, Matthews et al. (2001) used a MS2 and MS360, Ploj et al. (2003) only used MS15 and MS360, and Murgatroyd et al. (2009) used non-disturbed and MS180 with mice. Our current paradigm met the goal of optimizing the differences between conditions and is consistent with the type of two group design that is currently gaining momentum in this field.

For almost a decade, it has been known that maternal care during early neurological development influences DNA methylation that is directly responsible for HPA reactivity to stress throughout the lifespan. Weaver et al. (2004), showed that offspring of low licking/grooming and arch-back nursing (LG-ABN) mothers had higher levels of GR DNA methylation, decreased expression of the GR gene, a heighted HPA stress response, and displayed more fear-like behavior. Since this pioneering study, many laboratories have demonstrated various alterations in DNA methylation in adulthood following early life stress. For example, early life stress has been associated with increased global methylation, as well as increased methylation at the regulatory region of serotonin transporter (5-HTT), and higher behavioral stress responses in female macaques (Kinnally et al., 2011). Early life stress has also been found to induce hypomethylation of the Avp enhancer in male mice with a subsequent increased HPA reactivity (Murgatroyd et al., 2009). Although the brain region, gene, and direction in which DNA methylation is altered by early life stress is diverse, the outcome tends to remain constant, with a hyperactive HPA stress response and/or increased behavioral stress reactivity in adulthood. Since an overactive HPA axis and early life stress are strongly associated with a higher risk for drug addiction, additional research is needed to investigate if early life stress mediates epigenetic factors influencing the reward network that may predispose the animal to a higher propensity toward drug intake.

Methyl CpG binding protein 2 is a methylated DNA binding protein that attracts histone deacetylases (HDACs) and is commonly associated with specific gene silencing and repression of transcription (Jones et al., 1998), although it may also act to mediate transcription on a genome wide manner as well (Skene et al., 2010). Interestingly, drug exposure mediates levels of MeCP2 in various brain regions and manipulating MeCP2 levels prior to drug exposure can affect the drugs rewarding properties (Cassel et al., 2006; Deng et al., 2010; Im et al., 2010). Therefore we investigated if early life stress mediated MeCP2 levels in the NAc core, a brain region associated with the initial rewarding effects of drugs of abuse (Taylor et al., 2013). We observed group differences in MeCP2 immunoreactivity in the NAc core, such that MS15 rats expressed significantly higher levels of MeCP2 compared to MS180 rats.

Our results suggest a difference in DNA methylation in the NAc; however, the precise gene(s) where methylation has occurred and is bound by MeCP2 was not determined. Previous studies have suggested that MS rats may have altered DA, NE, and 5-HT function and GABA and glutamate levels in the NAc (Hall et al., 1999; Matthews et al., 2001; Romano-López et al., 2012). It has also been demonstrated that NAc protein expression is extensively changed after both MS and METH exposure (Dimatelis et al., 2012b). Therefore, the difference in methylated DNA may be associated with any number of genes involved in these systems in the NAc, and identification of methylated genes is worthy of further investigation. It is important to note that Romano-López et al. (2012) did not find a difference in MeCP2 levels in the NAc between their separated and non-separated pups using immunoblotting techniques. Thus, quantification by immunohistochemistry may not reveal the same results as by immunoblotting. Additionally, the differences in separation procedures and drug exposure potentially played a role in these contrary results.

The negative correlation between active lever presses and MeCP2 immunoreactivity in the NAc fits with Deng et al.’s (2010) study in which MeCP2 in the NAc had an inverse relationship with amphetamine CPP. This data warrants future directions in order to explicate this relationship, for example, additional studies are needed to determine the influence of rearing condition on MeCP2 levels in the NAc in drug-naïve animals as well as the influence of varying levels of METH exposure. Also worthy of future studies is the possibility that an enriched environment (EE) during adolescence could reverse the detrimental effects of MS on METH SA in adulthood and if it has a mediating effect on MeCP2 levels in the NAc. EE during an abstinence phase of cocaine showed protective effects to cue-induced reinstatement (Thiel et al., 2009) and reduced CPP to cocaine (Solinas et al., 2010). More recently, it was demonstrated that EE during different developmental time points can protect against METH SA acquisition and cue-induced reinstatement (Lü et al., 2012).

Few studies have investigated the effect of MS on drug relapse paradigms yet, there is little data that suggests early life stress may increase relapse vulnerability (Neisewander et al., 2012). Contrary to existing literature and our predictions that MS would influence extinction rates and cue-induced reinstatement, we failed to detect an effect. It is possible that we may have detected an extinction or reinstatement effect if the rats were trained on a progressive ratio or a higher FR of reinforcement since these schedules produce higher response rates. On the other hand, failing to find an effect may be indicative that rearing condition only influenced the initial rewarding or reinforcing effects of METH as opposed to the subsequent course of addiction, abstinence, and relapse. Also, we only tested for cue-induced reinstatement, future research is necessary to determine group differences in stress and drug induced reinstatement.

In summary, we observed that early life stress in the form of extended MS produced an increased vulnerability to adult METH SA in adult male rats or that a minimal daily MS led to resilience in adult METH SA. Increases in METH intake were paralleled by decreased MeCP2 immunoreactivity in the NAc core. Surprisingly, extinction and cue-induced reinstatement were unaffected by MS. These results suggest the possibility that early life stress may contribute to vulnerability toward METH intake. Further studies are needed to establish a contributory role for changes in MeCP2 levels in the NAc core or other brain regions in these behavioral effects.

## Conflict of Interest Statement

The authors declare that the research was conducted in the absence of any commercial or financial relationships that could be construed as a potential conflict of interest.
